# Exploiting the Anti-Biofilm Effect of the Engineered Phage Endolysin PM-477 to Disrupt In Vitro Single- and Dual-Species Biofilms of Vaginal Pathogens Associated with Bacterial Vaginosis

**DOI:** 10.3390/antibiotics11050558

**Published:** 2022-04-22

**Authors:** Joana Castro, Lúcia G. V. Sousa, Ângela França, Lenka Podpera Tisakova, Lorenzo Corsini, Nuno Cerca

**Affiliations:** 1 Centre of Biological Engineering (CEB), Laboratory of Research in Biofilms Rosário Oliveira (LIBRO), Campus de Gualtar, University of Minho, 4710-057 Braga, Portugal; joanacastro@ceb.uminho.pt (J.C.); luciasousa@ceb.uminho.pt (L.G.V.S.); afranca@ceb.uminho.pt (Â.F.); 2 LABBELS–Associate Laboratory, Braga/Guimarães, Portugal; 3 BioNTech R&D (Austria) GmbH, Vienna Biocenter, 1110 Wien, Austria; lenka.tisakova@phagomed.com (L.P.T.); lorenzo.corsini@phagomed.com (L.C.)

**Keywords:** bacterial vaginosis, *Gardnerella vaginalis*, *Fannyhessea vaginae*, *Prevotella bivia*, antimicrobial resistance, alternative therapies, endolysin, anti-biofilm

## Abstract

Bacterial vaginosis (BV) is the most frequent vaginal infection in women of reproductive age. It is caused by the overgrowth of anaerobic vaginal pathogens, such as *Gardnerella vaginalis*, *Fannyhessea vaginae*, and *Prevotella bivia*, which are vaginal pathogens detected during the early stages of incident BV and have been found to form multi-species biofilms. Treatment of biofilm-associated infections, such as BV, is challenging. In this study, we tested the role of an investigational engineered phage endolysin, PM-477, in the eradication of dual-species biofilms composed of *G. vaginalis*–*F. vaginae* or *G. vaginalis*–*P. bivia*. Single-species biofilms formed by these species were also analysed as controls. The effect of PM-477 on biomass and culturability of single- and dual-species biofilms was assessed in vitro using a microtiter plate assay, epifluorescence microscopy, confocal laser scanning microscopy, and quantitative PCR. The results showed that PM-477 was particularly effective in the disruption and reduction of culturability of *G. vaginalis* biofilms. In dual-species biofilms, PM-477 exhibited lower efficiency but was still able to selectively and significantly eliminate *G. vaginalis.* Since polymicrobial interactions have been shown to strongly affect the activity of various antibiotics, the activity of PM-477 in dual-species biofilms is a potentially promising result that should be further explored, aiming to completely eradicate multi-species biofilms associated with BV.

## 1. Introduction

Bacterial vaginosis (BV) is the worldwide leading vaginal bacterial infection identified in women of childbearing age [1] but remains one of the most intriguing and controversial challenges in modern-day clinical microbiology [2]. Although some women affected by BV are asymptomatic, approximately 50% of women experience a spectrum of symptoms, including vulvovaginal itching, burning, irritation, and abnormal vaginal odour and discharge [1]. BV is also associated with serious gynaecological and obstetric complications, including abortion [3], preterm birth [4], pelvic inflammatory disease, infertility, and endometritis [5]. Furthermore, BV can increase the risk of contracting sexually transmitted infections, including human immunodeficiency virus [6].

BV etiology is yet not fully understood, but one key hypothesis proposes that *Gardnerella* spp. is responsible for initiating the formation of the biofilm on vaginal epithelial cells that latter become a scaffold for other BV-associated species [7]. *Fannyhessea vaginae* (*F. vaginae*, previously known as *Atopobium vaginae* [8]) is one of the bacterial species that is often associated with the *Gardnerella* spp.-mediated biofilms [9]. *F. vaginae* has been associated with the presence of clue cells, vaginal discharge in women with BV, and elevated vaginal pH [10]. Importantly, the association between *F. vaginae* and *Gardnerella* spp. has been proposed as an important reason for therapeutic failures and high recurrence rates [11,12,13]. In addition, it was also described that high vaginal loads of *F. vaginae* in combination with *Gardnerella* spp. are associated with late miscarriage and premature birth [11]. Another bacterial species that has been associated with BV is *Prevotella bivia* (*P. bivia*) [7,14]. The virulence potential of *P. bivia* in BV was derived from studies that associated its colonization with preterm birth, endometritis, and other uterine pathologies [15,16,17]. Interestingly, Muzny and coworkers observed that the relative abundance of *P. bivia* was the first to rise above baseline prior to incident BV, followed shortly thereafter by an increase in the relative abundance of *Gardnerella* species. These authors proposed that the synergism between *P. bivia* and *Gardnerella* spp. might be an important event prior to BV [18].

The polymicrobial nature of BV biofilm is thought to protect BV-associated species when exposed to antibiotics, often making them ineffective and causing recurrent episodes [19,20]. In addition, the frequent ineffectiveness of standard antibiotics against BV has also been related to high rates of bacterial resistance [21,22]. Consequently, in an attempt to overcome BV treatment failure, an engineered endolysin (EL) has been proposed as a potentially promising and effective approach to treat this infection. Notably, Landlinger and colleagues demonstrated the efficacy and specificity of a novel investigational engineered EL, PM-477, to completely disrupt the *Gardnerella*-dominated biofilm in vaginal swabs from patients with BV [23]. However, since we recently showed that the presence of *F. vaginae* in multi-species biofilms enhances the tolerance of *G. vaginalis* to clindamycin and metronidazole [24], it is important to determine how multi-species interactions can affect the antimicrobial potential of this EL. Thus, in this study, we aimed to investigate the antimicrobial effect of PM-477 using an in vitro, dual-species biofilm model composed of *G. vaginalis* and *F. vaginae* and *G. vaginalis* and *P. bivia*.

## 2. Results

### 2.1. Quantification of the Biomass of Single- and Dual BV-Associated Biofilms

**Figure 1:**
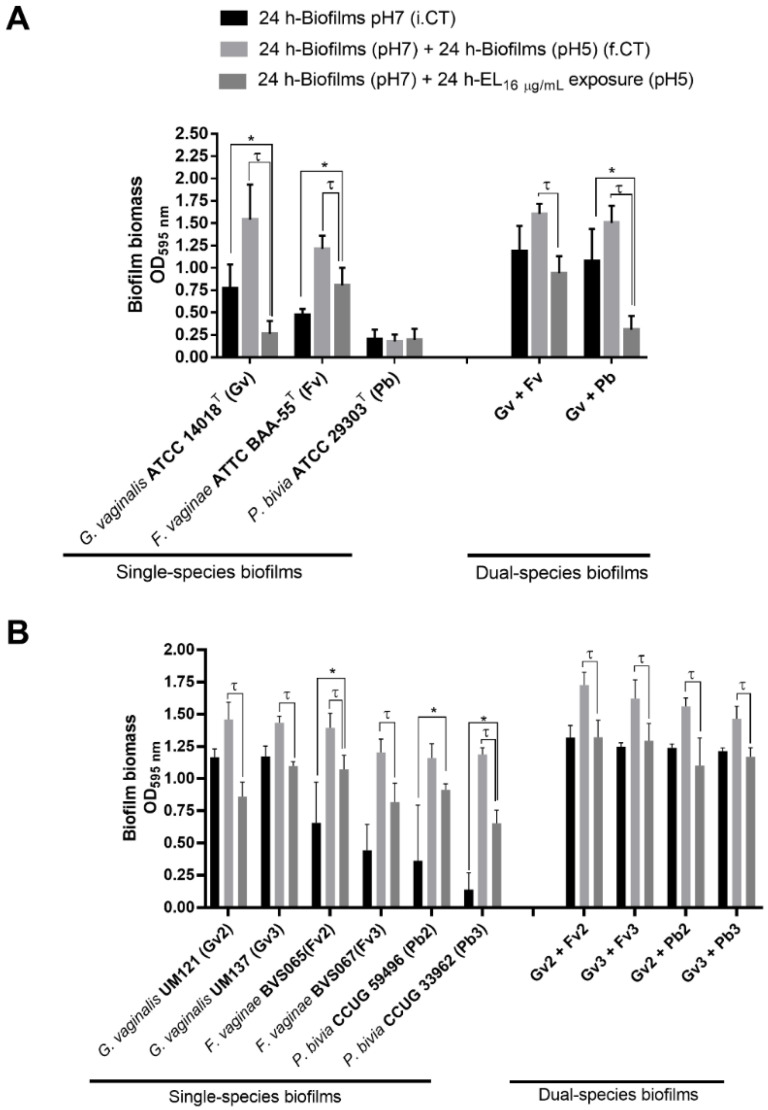
Effect of EL on single- and dual-species BV-associated biofilms. (**A**) PM-477 impact on single- and dual-species biofilms biomass constituted by *G. vaginalis*, *F. vaginae*, or *P. bivia* type strains as assessed by crystal-violet-staining assay. (**B**) PM-477 impact on single- and dual-species biofilm biomass constituted by *G. vaginalis*, *F. vaginae*, or *P. bivia* clinical isolates as assessed by crystal-violet-staining assay. Values are significantly different for * i.CT vs. PM-477 and τ f.CT vs. PM-477 (two-way ANOVA and Sidak’s multiple comparisons test, *p* < 0.05). Abbreviations: *Gardnerella vaginalis* (Gv); *Fannyhessea vaginae* (Fv); *Prevotella bivia* (Pb); optical density (OD); initial control at pH7 before the medium replacement (i.CT); final control at pH 5 after incubation with fresh medium (f.CT).

Although single-species biofilms are not a natural occurrence in BV, they were included in this study for comparative purposes. Interestingly, as shown in Figure 1A, biomass reduction after the PM-477 challenge was only observed for *G. vaginalis* biofilms when compared to pre-challenge levels (henceforth, referred to as initial control [i.CT]). However, in *F. vaginae* biofilms, although no reduction of the initial biofilm biomass occurred after the PM-477 challenge, biomass growth from 24 to 48 h was reduced, suggesting that PM-477 interfered with biomass accumulation. As shown before [25], *P. bivia* single-species biofilms formed very little biomass, and no effect was observed after the PM-477 challenge. When comparing the PM-477 effect on the dual-species biofilms, we observed a significant reduction in the biomass (as compared with the i.CT) only in the *G. vaginalis*–*P. bivia* consortium.

To confirm if this was strain-specific, in this preliminary screening, we repeated the same experiment with two additional strains of each tested species (Figure 1B). Interestingly, the non-ATCC *P. bivia* strains used in this confirmatory experiment were able to form thicker single-species biofilms. When using these strains, the PM-477 challenge resulted in biofilm accumulation reduction (from 24 to 48 h), similar to *F. vaginae* strains. Again, biomass reduction in comparison with the i.CT only occurred for *G. vaginalis*, but on the new strains this reduction was not statistically significant. Interestingly, in all dual-species consortia, PM-477 prevented biomass accumulation (from 24 to 48 h) but was not able to reduce the existing biomass.

### 2.2. Impact of PM-477 on Biofilm Cell Culturability

**Figure 2:**
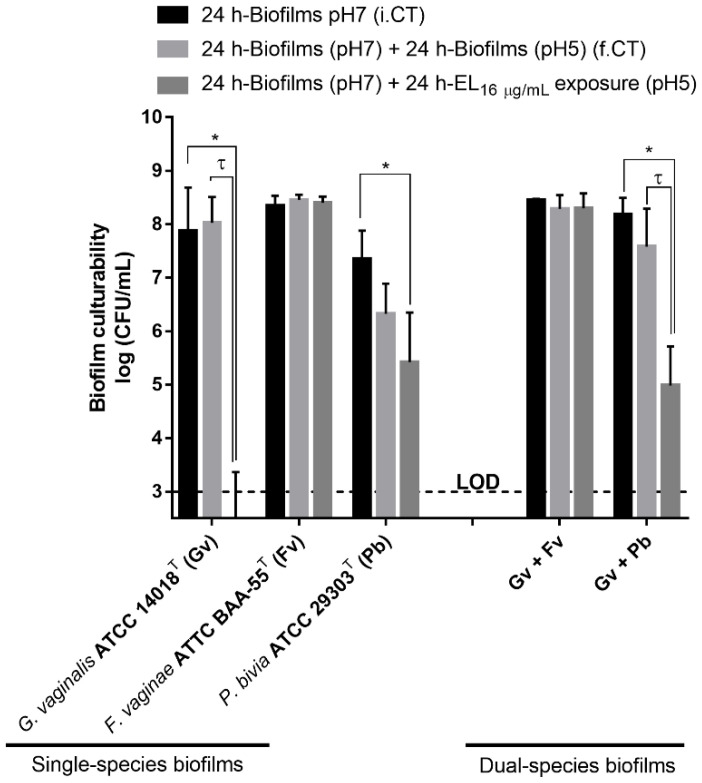
Effect of EL on single- and dual-species BV-associated biofilms constituted by *G. vaginalis*, *F. vaginae*, or *P. bivia* type strains. Cell culturability was determined by CFU method. Values are significantly different for * i.CT vs. PM-477 and τ f.CT vs. PM-477 (two-way ANOVA and Sidak’s multiple comparisons test, *p* < 0.05). Abbreviations: *Gardnerella vaginalis* (Gv); *Fannyhessea vaginae* (Fv); *Prevotella bivia* (Pb); Endolysin (EL); initial control at pH 7 before the medium replacement (i.CT); final control at pH 5 after incubation with fresh medium (f.CT); limit of detection (LOD).

In order to confirm the results obtained from crystal violet (CV)-staining screening, we also assessed if biofilm biomass reduction was reflected in the decrease of the concentration of culturable cells. As shown in Figure 2, PM-477 has robust antimicrobial activity only against *G. vaginalis* (approximately 5 log-reduction taking into account the limit of detection), confirming the previous observations of the antimicrobial spectrum of action [23]. Of note, *P. bivia* culturability was slightly reduced at a lower pH. When comparing the PM-477 effect on dual-species biofilms, the colony-forming unit (CFU) data confirmed that no effect was observed in the *G. vaginalis*–*F. vaginae* consortium but some antimicrobial effect was observed in the *G. vaginalis*–*P. bivia* biofilm (approximately 3 log-reduction).

### 2.3. PM-477 Influences the Performance of PNA FISH

**Figure 3:**
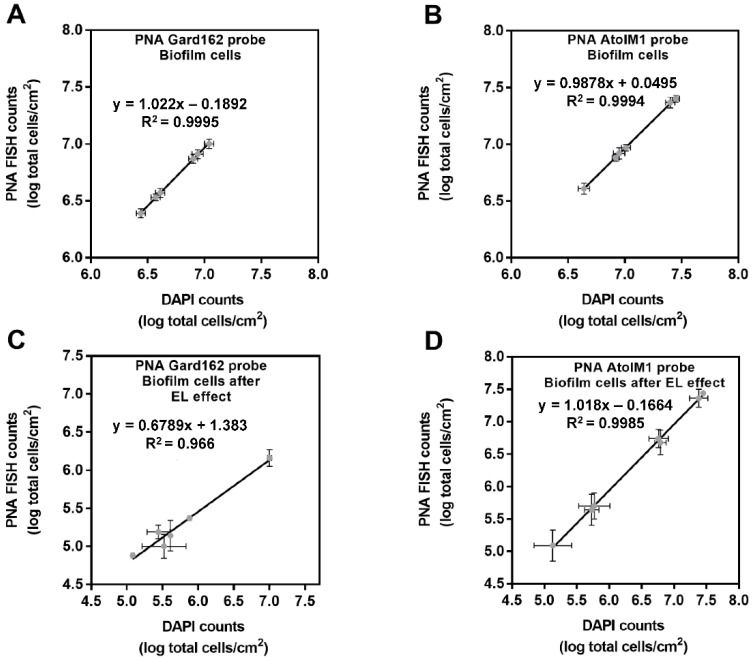
Correlation between PNA FISH counts and the DAPI counts for single-species biofilms. (**A**) Correlation between PNA Gard162 probe counts and the DAPI counts for single-species *G. vaginalis* biofilm cells. (**B**) Correlation between PNA AtoITM1 probe counts and the DAPI counts for single-species *F. vaginae* biofilm cells. (**C**) Correlation between PNA Gard162 probe counts and the DAPI counts for single-species *G. vaginalis* biofilm cells after PM-477 (16 µg/mL) challenge. (**D**) Correlation between PNA AtoITM1 probe counts and the DAPI counts for single-species *F. vaginae* biofilm cells after PM-477 (16 µg/mL) challenge. Each data point represents the mean ± s.d. from three independent assays. For each assay, 13 fields were randomly acquired in each sample and the number of bacteria per image was counted using *ImageJ* software. Abbreviations: endolysin (EL); peptide nucleic acid fluorescence in situ hybridization (PNA FISH); 4′,6-diamidino-2-phenylindole (DAPI).

Taking into consideration the single-species susceptibility to EL, it is reasonable to hypothesize that in dual-species biofilms exposed to EL, an enrichment of the tolerant species could occur. To assess this, we first carried out an experiment wherein we aimed to discriminate the percentage of each bacterial species in the tested dual-species biofilms after PM-477 exposure. Our first attempt was performed using a previously developed and validated peptide nucleic acid fluorescence in situ hybridization (PNA FISH) method [26]. As described before, for accurate quantification, it is required to determine the efficiency of each PNA FISH probe against a non-specific fluorescence dye [27]. As such, we first assessed if PM-477 interferes with the efficiency of PNA probes. To achieve this, we compared the data obtained from PNA FISH and 4′,6-diamidino-2-phenylindole (DAPI) counts for both *G. vaginalis* and *F. vaginae* cells disrupted from single-species biofilms with and without PM-477 exposure, as shown in Figure 3.

Interestingly, we observed that PNA Gard162 probe efficiency was significantly affected after the PM-477 challenge, while this did not occur for the AtoITM1 probe (Table 1). Taking into consideration that a reduction in PNA probe efficiency is expected to occur when cells lose viability [28], these results further demonstrate the specificity and the antimicrobial potential of the tested PM-477 against *G. vaginalis*.

### 2.4. Prevalence of Bacterial Populations in the Dual-Species Biofilms before and after PM-477 Challenge

**Figure 4:**
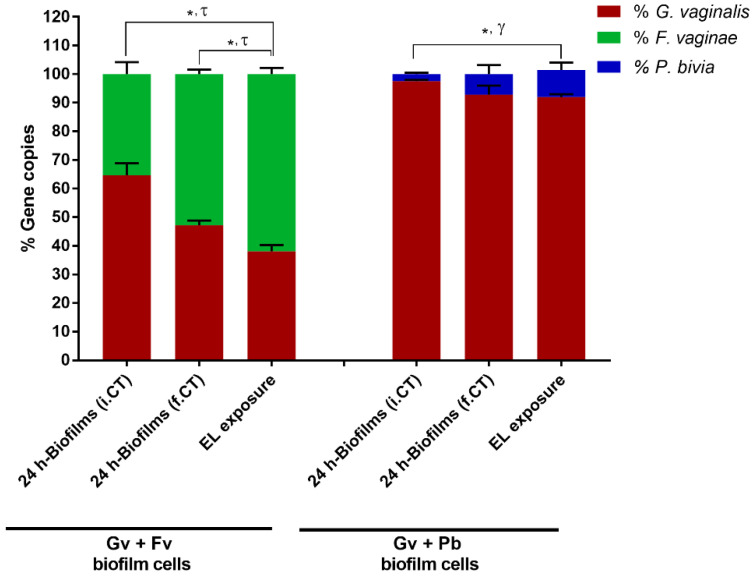
Determination of the percentage distribution of each bacterial species in dual-species biofilms prior and after PM-477 treatment, by qPCR. Statistically significant differences were observed between the percentage of gene copies of * Gv in controls vs. EL-exposed biofilms; τ Fv controls vs. EL-exposed biofilms, γ Pb controls vs. EL-exposed biofilms (two-way ANOVA and Sidak’s multiple comparisons tests, *p* > 0.05). Abbreviations: *Gardnerella vaginalis* strain ATCC 14018^T^ (Gv); *Fannyhessea vaginae* strain ATTC BAA-55^T^ (Fv); *Prevotella bivia* strain ATCC 29303^T^ (Pb); endolysin (EL); initial control at pH 7 before the medium replacement (i.CT); final control at pH 5 after incubation with fresh medium (f.CT).

To overcome the limitation of microscopic experiments described above, we analysed the bacterial composition in the dual-species biofilms through quantification of specific gene copy numbers by qPCR. As shown in Figure 4, 24 h dual-species biofilms were mainly composed of *G. vaginalis*, as reported previously [29]. Interestingly, there was a slight enrichment of both *F. vaginae* and *P. bivia* after the PM-477 challenge, which is not unexpected since these two species are tolerant to PM-477 action. Importantly, in line with previous findings [24], evidence of a protective effect from either *F. vaginae* or *P. bivia* was noted by the still significant number of *G. vaginalis* present. While qPCR has its limitations, there is no expectation that DNA extraction and qPCR efficiency would be influenced by the PM-477 treatment. However, qPCR amplification of gDNA does not unequivocally distinguish between viable and non-viable cells in in vitro biofilms [30,31]. To overcome this limitation, we disrupted the dual-species biofilms formed after PM-477 treatment and assessed the presence of viable *G. vaginalis* by epifluorescence microscopy, using the Gard162 PNA probe. As shown in Supplementary Figure S1, both dual-species consortia contain viable *G. vaginalis*, further supporting the conclusion that PM-477 activity is reduced in the presence of other BV-associated species.

### 2.5. Indirect Assessment of PM-477 Activity in Biofilm Structure

**Figure 5:**
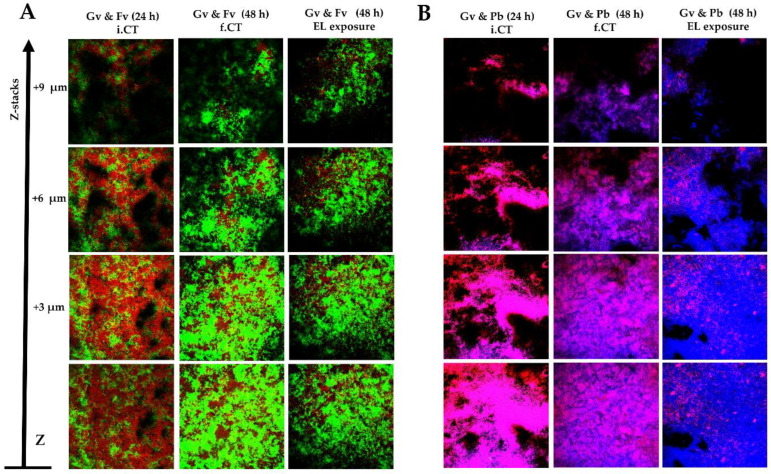
Representative CLSM image showing the organization of the dual-species BV-associated biofilms. (**A**) An example data set on the organization of Gv and Fv dual-species biofilms. (**B**) An example data set on the organization of Gv and Pb dual-species biofilms. Gv and Fv cells were differentiated by hybridization with PNA Gard162 (red/purple color when coupled with DAPI) and AtoITM1 probes (green color), respectively, while Pb and non-hybridized Gv cells were differentiated by DAPI (blue color). Abbreviations: *Gardnerella vaginalis* strain ATCC 14018^T^ (Gv); *Fannyhessea vaginae* strain ATTC BAA-55^T^ (Fv); *Prevotella bivia* strain ATCC 29303^T^ (Pb); endolysin (EL); initial control at pH7 before the medium replacement (i.CT); final control at pH5 after incubation with fresh medium (f.CT).

Our previous experiments found reduced antimicrobial activity against *G. vaginalis* when PM-477 is used in in vitro dual-species biofilms. Nevertheless, at least in the *G. vaginalis*–*P. bivia* consortium, some reduction in CV quantification was observed. Due to the limitations of the quantification of CV staining in multi-species biofilms, we analysed biofilm structure by confocal laser scanning microscopy (CLSM). As shown in Figure 5, relative fluorescence was strongly affected by the PM-477 treatment, with the specific PNA Gard162 probe being dramatically reduced, although not completely eradicated. This is in line with all previous observations described above. Furthermore, we did not observe significant alterations in the biofilms 3D structure after the PM-477 challenge, with clusters of cells and several layers of biomass being present in all tested situations.

## 3. Discussion

Although the current antibiotics used against BV are somewhat effective, the management of this infection continues to be challenging [32,33]. The presence of a multi-species BV biofilm adhered to vaginal epithelial cells is considered one of the major factors responsible for the treatment failure, which may be explained by the low efficacy of antibiotics on bacteria growing as biofilms [34,35]. Furthermore, the use of antibiotics can promote selective destruction of beneficial lactobacilli, which are thereby prevented from re-establishing a protective vaginal environment [36]. As such, the antibiotic-centered treatment for BV is associated with a recurrence of approximately 60% within six months, resulting in a serious problem for women’s gynaecological and obstetric health [20]. To overcome this problem, alternative approaches to existing antibiotics are being currently pursued [22,23,30,37]. In this study, we further explored the potential of an EL, called PM-477, against BV. Recently, Landlinger and colleagues have shown that PM-477 showed minimum inhibitory concentrations of 0.13 to 8 µg/mL against *Gardnerella* spp. and did not affect beneficial lactobacilli [23].

Herein, we selected the concentration of 16 µg/mL to assess its ability to eradicate in vitro single- and dual-species biofilms, formed of *G. vaginalis* and *F. vaginae* or *P. bivia*, which are species associated with incident BV.

PM-477 has previously been described as an antimicrobial agent against *Gardnerella* spp. planktonic cells and is also known to possess high selectivity and effectiveness in eliminating *Gardnerella* spp. in clinically derived samples of women with BV [23]. Furthermore, a recent study by Latka and colleagues confirmed the high antimicrobial potential of PM-477 EL, which demonstrated a reduction of up to 99.4% of the viability of *Gardnerella* spp. present in vaginal samples from women with BV [31]. In our study, we found that in addition to this antimicrobial activity, PM-477 is also active against *G. vaginalis* in vitro biofilms, thus reducing their culturability and to some extent their biomass.

We recently demonstrated that evaluation of multi-species biofilms using CV staining should be carried out with caution, since different species contribute differently to CV quantification, and this can lead to biased analysis [38]. As such, herein, different approaches were used to assess the anti-biofilm effect of PM-477. In spite of being able to inhibit biomass accumulation from 24 to 48 h, PM-477 had no detectable effect on the culturability of *F. vaginae*.

This is worth investigating further since *F. vaginae* is strongly associated with BV and co-colonization with *G. vaginalis* [21,39] and has been suggested as a potential contribution to the failure of BV treatment [12,13]. Furthermore, our FISH, CLSM, and qPCR data suggest that PM-477 strongly affects dual-species biofilms, even if some *G. vaginalis* remain intact and viable after PM-477 treatment. It is accepted that the biofilm can serve as a protective barrier, and its thickness and chemical composition can limit the perfusion and/or activity of antimicrobial compounds [40], an effect that might be over-represented in the in vitro biofilms studied here compared to the typically thinner, physiological BV-associated biofilm [21,39]. This specific feature may lead to the protection of a minor fraction of cells in the in vitro biofilms, which, in most cases, will be further able to reinitiate biofilm formation and, as such, contribute to recurrent infections. However, the possible implications of these biofilm cells on possible BV recurrence require further study.

## 4. Materials and Methods

### 4.1. Bacterial Strains and Culture Conditions

*G. vaginalis* strain ATCC 14018^T^, *F. vaginae* strain ATTC BAA-55^T^, and *P. bivia* strain ATCC 29303^T^ were used in this study. For the biofilm experiments (when biofilm biomass was assessed), two additional strains of *G. vaginalis*, namely UM121 and UM137, two additional strains of *F. vaginae*, namely BVS065 and BVS067, and two additional strains of *P. bivia*, namely CCUG 59496 and CCUG 33962 were used (see Supplementary Table S1). Bacteria were grown in New York City III broth (NYC III) supplemented with 10% (*v*/*v*) inactivated horse serum (Biowest, Nuaillé, France), as described before [25], for 24 h at 37 °C under anaerobic conditions (AnaeroGen Atmosphere Generation system, Oxoid, UK), previously shown to be the optimal conditions to grow single-species biofilms of the selected bacterial species [25].

### 4.2. Preparation of PM-477

The engineered EL PM-477 was produced, as previously described [23], followed by His-tag cleavage. Briefly, PM-477 was recombinantly expressed in *Escherichia coli* BL21 (DE3). Protein purification was performed by affinity chromatography on a nickel–nitrilotriacetic acid (Ni–NTA) HISTrap column. The protein was eluted with 50 mM MES (Carl Roth, Karlsruhe, Germany, pH 7, 150 mM NaCl (Carl Roth), 150–500 mM imidazole fractions (two-fold dilutions). Subsequently, the N-terminal His-tag was cleaved off by digestion with 1:100 *w*/*w* 3C protease. The removed tag and the protease were separated from PM-477 EL by anion exchange chromatography. The untagged protein was concentrated and dialyzed against MES storage buffer (50 mM MES pH 5.5, 200 mM NaCl, 8 mM MgSO_4_ (Sigma Aldrich, St. Louis, MO, USA). Protein concentration was determined at OD 260/280 nm or by using the Pierce^TM^ BCA (bicinchoninic acid) protein assay kit (Thermo Fisher Scientific, Waltham, MA, USA). Purified PM-477 aliquots of 1000 µL and at a concentration of 1.4 mg/mL were stored at −80 °C until further use.

### 4.3. Dual-Species Biofilm Formation and EL Challenge

Cultures of *G. vaginalis*, *F. vaginae*, and *P. bivia* were adjusted to a concentration of 1.0 × 10^7^ CFU/mL in NYC III at pH 7, as determined before [41]. For dual-species biofilm formation, either *F. vaginae* or *P. bivia* was co-incubated with both *G. vaginalis* in 24-well tissue culture plates (Braine-l’Alleud, Belgium) for 24 h at 37 °C under anaerobic conditions, as mentioned above. Afterwards, the medium covering the biofilms was carefully removed and, the biofilms from each well were exposed to engineered phage EL, called PM-477 [23], at 16 µg/mL diluted in NYC III at pH 5, due to the optimal lytic activity in an acidic environment, for further anaerobic incubation at 37 °C for an additional 24 h. Single-species biofilms were grown as controls. Furthermore, two other types were controls where biofilms were not subjected to antimicrobial treatment: (i) 24 h dual-species biofilms at pH 7 and (ii) 48 h dual-species biofilms at pH 5. The biofilms were then washed once with 1 mL 1× phosphate-buffered saline (PBS), being set for the following experiments. All assays were repeated independently at least three times with technical replicates.

### 4.4. Quantification of Bacterial Biofilm by Crystal Violet (CV) Staining

Biofilms biomass was quantifyed using the crystal violet (CV) method, as described before [42]. Briefly, biofilms were fixation with 100% (*v*/*v*) methanol (Thermo Fisher Scientific) for 20 min and then were stained with CV solution at 1% (*v*/*v*) (Merck, Darmstadt, Germany) for another 20 min. Biofilms were washed twice with phosphate-buffered saline (PBS). The CV stained was then recovered from the biofilm with the addition of 33% (*v*/*v*) acetic acid (Thermo Fisher Scientific), that was then transferred to a new plate before optical density (OD) at 595 nm was assessed. These assays were repeated three times on separate days, with four technical replicates assessed each time.

### 4.5. Enumeration of Total Culturable Bacteria in the Biofilm Using the CFU Counting Approach

Following formation (for 24 h or 48 h), biofilms were carefully washed with 0.9% (*w*/*v*) NaCl, and 1 mL of NYC III was added into each well. The biofilms were then scraped, and a pool of two different wells was obtained for each condition. Then, for each condition, serial dilutions ranging from 10^−1^ to 10^−6^ were performed on the biofilm pool suspensions diluted in 0.9% (*w*/*v*) NaCl. After homogenization, 10 µL of each dilution was spread onto Columbia Base Agar (CBA) plates supplemented with 5% (*v*/*v*) horse defibrinated blood and then incubated at 37 °C for 72 h under anaerobic conditions. After this period, the colonies were counted in the appropriate dilution and the bacterial concentration was calculated accordingly. These assays were repeated three times on separate days, with two technical replicates assessed each time.

### 4.6. Impact of PM-477 on the Efficiency of PNA FISH Probes

Prior to microscopic analysis, we assessed if the PM-477 challenge with *G. vaginalis* and *F. vaginae* single-species biofilms affected the performance of the PNA FISH method. As such, we determined the efficiency of the PNA probes by using several dilutions of *G. vaginalis* and *F. vaginae* single-species biofilm cells without and after 24 h of the PM-477 challenge, correlating the DAPI with PNA FISH counts, as previously described [41]. In brief, biofilms aliquots were first fixed on epoxy-coated microscope glass slides (Thermo Fisher Scientific). Then, a PNA probe specific for *G. vaginalis* (Gard162) [43] or for *F. vaginae* (AtoITM1) [44] was added to each well. At the end of the hybridization procedure, an additional staining step was done, covering each glass slide with DAPI (2.5 μg/mL). Microscopic visualization was performed using filters capable of detecting the PNA Gard162 probe (BP 530-550, FT 570, and LP 591 sensitive to the Alexa Fluor 594 molecule attached to the Gard162 probe), the PNA AtoITM1 probe (BP 470-490, FT500, and LP 516 sensitive to the Alexa Fluor 488 molecule attached to the AtoITM1 probe), and DAPI (BP 365-370, FT 400, LP 42). For each sample, thirteen fields were randomly acquired. The number of bacteria was counted using ImageJ software [45]. These assays were repeated three times on separate days.

### 4.7. Effect of PM-477 on the Bacterial Composition of Dual-Species Biofilms Assessed by Quantitative PCR (qPCR) Quantification

Genomic DNA (gDNA) was extracted using the DNeasy Ultraclean Microbial Kit (QIAGEN, Hilden, Germany), following the manufacturer’s instructions with some minor modifications. Briefly, bacterial samples were centrifuged at 11,000× *g* for 5 min, the supernatant was carefully removed, and the pellet was frozen at −20 °C overnight. This step increased the DNA yield up to two-fold. The cells were lysed in a BeadBug 6 Microtube Homogenizer (Benchmark Scientific, Sayreville, NJ, USA) using 2 × 3 cycles of 30 s at 4350 rpm. Samples were kept on ice between the two cycles. To evaluate the efficiency and variability of gDNA extraction between samples, 10 µL of luciferase complementary DNA (cDNA), obtained as previously described [46], was added to each sample before proceeding with gDNA isolation. gDNA was eluted in 50 µL of DNase-free water. To normalise the bacterial load with extracted gDNA quantity, a calibration curve was generated with gDNA isolated from pure bacterial cultures with concentrations ranging from 5 × 10^6^ to 1 × 10^9^ cells/mL, as determined by OD and flow cytometry [29], was performed. Specific primers were designed with CLC genomics workbench version 21 (QIAGEN), and primer specificity was first confirmed using Primer-BLAST and then experimentally determined by qPCR. Primers are described in Supplementary Table S2. Before performing the qPCR, gDNA samples were diluted 10× in DNase-free water. Each qPCR reaction included 2 µL of diluted gDNA, 5 µL of Xpert Fast SYBR (Grisp, Porto, Portugal), 1 µL of primers mixture (at 10 µM), and 2 µL of water. All samples were analysed in triplicate. In order to assess for reagent contamination, non-template controls were performed. To evaluate the efficiency of gDNA extraction and to calibrate data between qPCR runs, a control was used by adding 2 µL of a 1:50 dilution of cDNA luciferase to each qPCR plate. The qPCR runs were performed in a CFX96TM thermal cycle (Bio-Rad, Hercules, CA, USA) with the following cycle parameters: 95 °C for 3 min, followed by 40 cycles of 95 °C for 5 s, and 60 °C for 20 s. At the end of the amplification sequence, a melt analysis was performed to ensure the absence of unspecific products and primer dimers. PCR amplification efficiency was determined from the slope of a standard curve. Bacterial load in each sample was interpolated from the averaged standard curves. All assays were repeated at least three independent times with three technical replicates.

### 4.8. Confocal Laser Scanning Microscopy Analysis of Biofilm Bacterial Distribution

Biofilms were formed on an 8-well chamber slide (Thermo Fisher Scientific™ Nunc™ Lab-Tek™, New York, NY, USA) at 37 °C for 48 h with the replacement of NYC III medium at 24 h of growth and the addition of EL under anaerobic conditions. For biofilms composed of *G. vaginalis* and *F. vaginae*, we used the PNA Gard162 and AtoITM1 probes, while for biofilms composed of *G. vaginalis* and *P. bivia*, we used the PNA Gard162 probe coupled to DAPI staining, as described above. The CLSM images were acquired in an Olympus™ FluoView FV1000 (Olympus) CLSM, using a 10 × objective. Microscopic visualization was performed using lasers capable of detecting the PNA Gard162 probe (Laser 559, excitation wavelength 559 nm, emission wavelength 618 nm, BA 575–675, sensitive to the Alexa Fluor 594 molecule attached to the Gard162 probe), the PNA AtoITM1 probe (Laser 488, excitation wavelength 488 nm, emission wavelength 520 nm, BA 505–540, sensitive to the Alexa Fluor 488 molecule attached to the AtoITM1 probe), and DAPI (Laser 405, excitation wavelength 405 nm, emission wavelength 461 nm, BA 430–470). Images were acquired with 512 × 512 resolution of each surface analyzed. All assays were repeated independently three times with two technical replicates.

### 4.9. Statistical Analysis

The data were analyzed using the statistical package GraphPad Prism version 6 (La Jolla, CA, USA) by two-way ANOVA (Sidak’s multiple comparisons tests) since the data follow a normal distribution according to Kolmogorov–Smirnov’s test. Values with a *p* < 0.05 were considered statistically significant.

## 5. Conclusions

In summary, we show that PM-477 is able to kill *G. vaginalis* in single- and dual-species biofilms, and despite a small reduction of antimicrobial activity of PM-477 in dual-species biofilms, this is a promising result to be further explored, since polymicrobial interactions have been shown to strongly affect antibiotic activity.

## Figures and Tables

**Table 1:** Percentage of efficiency of PNA probes without and with PM-477 (16 µg/mL) exposure.

Condition	Probe Efficiency (%)
*G. vaginalis* biofilm	92.08 *
*G. vaginalis* biofilm after PM-477 exposure (16 µL/mL)	38.90 *
*F. vaginae* biofilm	91.59
*F. vaginae* biofilm after PM-477 exposure (16 µL/mL)	89.28

Note: values are significantly different for * *G. vaginalis* biofilm cells without and with EL challenge two-way ANOVA and Sidak’s multiple comparisons test, *p* < 0.05. Abbreviations: peptide nucleic acid (PNA).

## Data Availability

The data presented in this study are available within the article and Supplementary Materials.

## Supplementary Materials

The following are available online at https://www.mdpi.com/article/10.3390/antibiotics11050558/s1, Figure S1: An example data set on the organization of the dual-species BV-associated biofilms by epifluorescence microscopy; Table S1: Bacterial strains used in this study; Table S2: Specific primers for the quantification of the bacterial species present in the dual-species biofilm.
