# Supplementary material for: Exploiting the Anti-Biofilm Effect of the Engineered Phage Endolysin PM-477 to Disrupt In Vitro Single- and Dual-Species Biofilms of Vaginal Pathogens Associated with Bacterial Vaginosis
Source: Antibiotics (Basel). 2022 Apr 22;11(5):558. doi: 10.3390/antibiotics11050558 (PMC9137943; doi:10.3390/antibiotics11050558)
Supplement: Supplementary file 1 [file antibiotics-11-00558-s001.zip › antibiotics-1638048-supplementary.pdf]

Supplementary Table S1. Bacterial strains used in this study.

Bacterial strain	Abbreviation
Gardnerella vaginalis strain ATCC 14018 T	Gv
Gardnerella vaginalis strain UM121	Gv2
Gardnerella vaginalis strain UM137	Gv3
Fannyhessea vaginae strain ATTC BAA-55T	Fv
Fannyhessea vaginae strain BVS065	Fv2
Fannyhessea vaginae strain BVS067	Fv3
Prevotella bivia strain ATTC 29303 T	Pb
Prevotella bivia strain CCUG 59496	Pb2
Prevotella bivia strain CCUG 33962	Pb3

Supplementary Table S2. Specific primers for the quantification of the bacterial species present in the dual-species biofilm.

Target genome	Genomic amplification region	Forward primer	Reverse primer	Amplicon	Amplification efficiency
Gardnerella vaginalis ATCC 14018	locus_tag=GAVG_1017 (product=glucose-6-phosphate isomerase)	CAACGGTATCCTGACCGTCT	CCTTGCAAAGGCAGTTAAGC	155 bp	82%
Fannyhessea vaginae FDAARGOS934	locus_tag=I6G91_00565 (product=PTS sugar transporter subunit IIB)	CCTCATGCAAAATGTGATGC	CCAAAACAGAAGCACGGAAT	211 bp	80%
Prevotella fusca JCM 17724[#]	locus_tag= ADJ77_02570 (product=endolytic transglycosylase MltG)	GGCTGATGAATATGCCTACGA	AAACACCATGTTGCGAATAGC	236 bp	85%
Exogenous control (Luciferase)	N/A	TACAACACCCCAACATCTTCGA	GGAAGTTCACCGGCGTCAT	67 bp	100%

[#]-Due to the lack of annotated *P. bivia* genomes at NCBI, we used a conservative region of a similar *Prevotella* species for primer design, which was later confirmed *in vitro.*

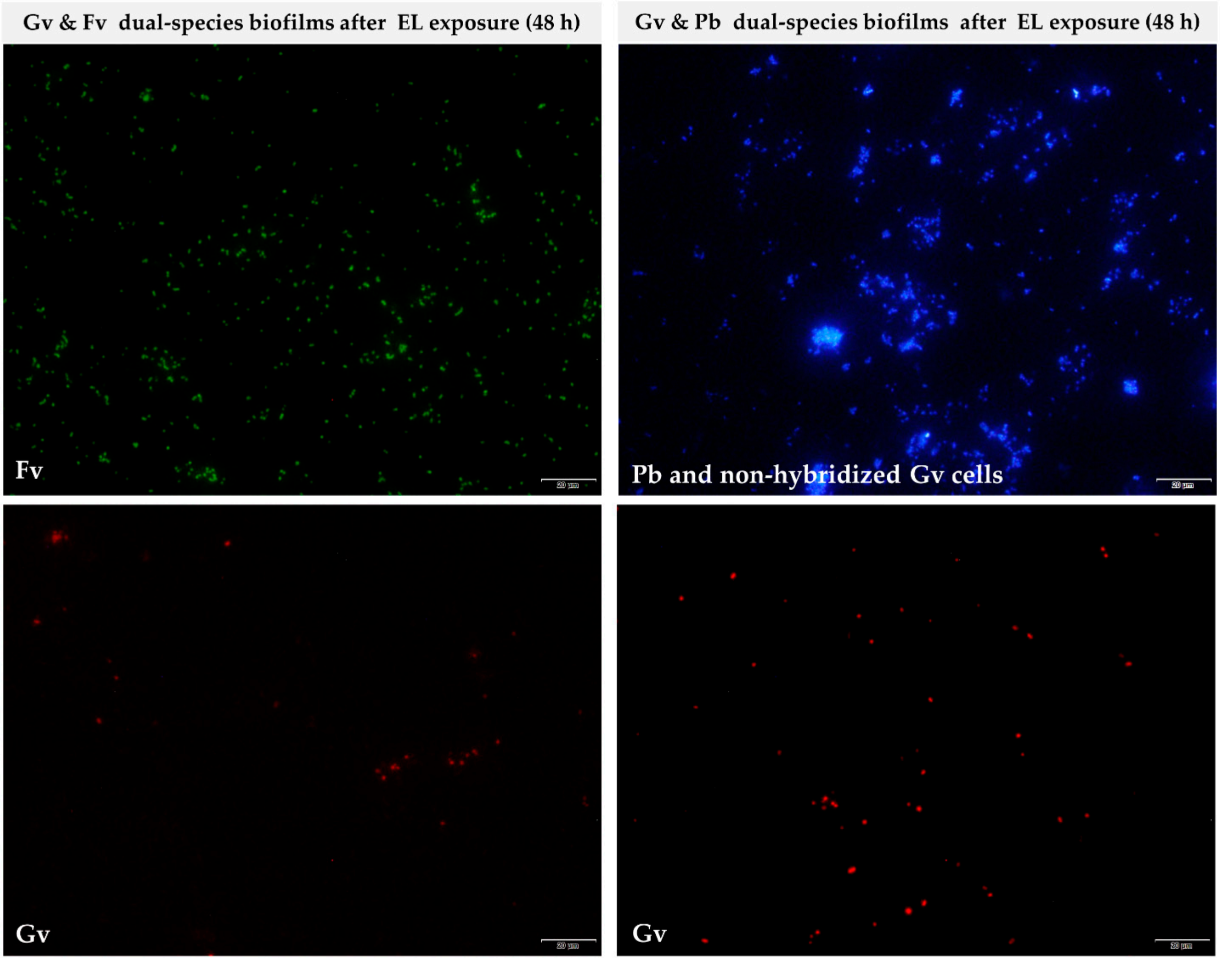

Supplementary Figure S1. An example data set on the organization of the dual-species BV-associated biofilms by epifluorescence microscopy. Gv and Fv cells were differentiated by hybridization with PNA Gard162 (red color) and AtoITM1 probes (green color), respectively, while Pb and non-hybridized Gv cells were differentiated by DAPI (blue color). Magnification of x400. Abbreviations: *Gardnerella vaginalis* strain ATCC 14018^{T} (Gv); *Fannyhessea vaginae* strain ATTC BAA-55^{T} (Fv); *Prevotella bivia* strain ATCC 29303^{T} (Pb); Endolysin (EL).
